# Accuracy of Invisalign® aligners in adult patients: a retrospective study of angular tooth movements

**DOI:** 10.1590/2177-6709.29.2.e2423237.oar

**Published:** 2024-05-20

**Authors:** Raquel Bueno MEDEIROS, Renata Faria SANTOS, Jose Augusto MENDES-MIGUEL, Eduardo Kant Colunga ROTHIER, Fausto Medeiros MENDES, Gladys Cristina DOMINGUEZ

**Affiliations:** 1 Universidade de São Paulo, Faculdade de Odontologia, Departamento de Ortodontia (São Paulo/SP, Brazil).; 2 Universidade Estadual do Rio de Janeiro, Faculdade de Odontologia, Departamento de Ortodontia (Rio de Janeiro/RJ Brazil).; 3 Private practice (Rio de Janeiro, Brazil).; 4 Universidade de São Paulo, Faculdade de Odontologia, Departamento de Odontopediatria (São Paulo/SP, Brazil) .

**Keywords:** Retrospective studies, Removable orthodontic appliance, Malocclusion, Tooth movement, Estudo retrospectivo, Aparelhagem ortodôntica removível, Má oclusão, Movimento dentário

## Abstract

**Objective:**

This retrospective study aimed to assess the predictability of Invisalign® aligners regarding rotational, mesio-distal and buccal-lingual tip movements.

**Methods:**

Two materials were included in the analysis - EX30, used until 2013; and SmartTrack, in current use. The study comprised 56 adult patients treated with Invisalign Comprehensive. Data sample were assessed on three sets of digital models; model 1 - initial, model 2 - predicted, and model 3 - achieved. Sixty reference points were marked in each dental arch, and two reference planes assisted the superimposition. The degree of rotation, mesio-distal and buccal-lingual tip was obtained via trigonometric calculations, through a previously published validated method. The accuracy of outcomes was compared according to the types of tooth movement and teeth groups,and the influence of predetermined variables on movement accuracy was also investigated.

**Results:**

Rotation and mesio-distal tip did not present any significant difference when comparing EX30 and SmartTrack groups. Only buccal-lingual tip presented a significant difference, incisor and canine groups treated with EX30 aligners presented an increase in accuracy (*p*= 0.007 and *p* = 0.007, respectively). For each additional degree planned for rotation movements, there was an increase of 0.35° in the discrepancy, and an increase of 0.40° and 0.41° for mesio-distal and buccal-lingual tip, respectively. EX30 and SmartTrack discrepancies were compared by multilevel linear regression.

**Conclusion:**

EX30 aligners reached higher accuracy for buccal-lingual tip in anterior teeth. However, for rotation and mesio-distal tip, SmartTrack and EX30 are similarly accurate. The total amount of planned movement has a significant impact on accuracy rates, with a decrease in accuracy for every additional degree.

## RESUMO

**Objetivo:**

Esse estudo retrospectivo teve como objetivo aferir a previsibilidade dos alinhadores Invisalign em relação aos movimentos de rotação e inclinações mesiodistal e vestibulolingual.

**Material e Métodos:**

Foram incluídos dois materiais nessa análise: EX30^®^, utilizado até 2013; e SmartTrack^®^, atualmente em uso. Esse estudo avaliou 56 pacientes tratados com Invisalign Comprehensive. Os dados foram avaliados em três pares de modelos digitais; modelo 1 - inicial, modelo 2 - planejado, e modelo 3 - alcançado. Foram marcados 60 pontos de referência em cada arcada, sendo a sobreposição realizada com auxílio de planos de referência. O grau de rotação e inclinações mesiodistal e vestibulolingual foi obtido por cálculos de trigonometria, usando um método validado publicado anteriormente. Os resultados foram comparados de acordo com os tipos de movimento dentário e grupos de dentes, também foi investigada a influência de variáveis predeterminadas na confiabilidade dos movimentos.

**Resultados:**

Os movimentos de rotação e inclinação mesiodistal não apresentaram diferença estatística, quando comparados os grupos EX30^®^ e SmartTrack^®^. Somente a inclinação vestibulolingual apresentou diferença estatisticamente significativa, sendo que os grupos de incisivos e caninos tratados com alinhadores EX30^®^ apresentaram um aumento na previsibilidade(*p*= 0,007 e *p*= 0,007, respectivamente). Para cada grau adicional planejado para movimento de rotação, ocorreu um aumento de 0,35° na discrepância, e aumentos de 0,40° e 0,41° para as inclinações mesiodistal e vestibulolingual, respectivamente. As discrepâncias entre EX30^®^ e SmartTrack^®^ foram comparadas por regressão linear multinível.

**Conclusão:**

Os alinhadores EX30^®^ alcançaram maior previsibilidade para a inclinação vestibulolingual em dentes anteriores. No entanto, para rotação e inclinação mesiodistal, SmartTrack^®^ e EX30^®^ apresentaram previsibilidade similar. A quantidade total de movimento planejado apresenta influência significativa nas taxas de previsibilidade, com diminuição na acurácia para cada grau adicional.

## INTRODUCTION

Invisalign^®^ (Align Technology, Santa Clara, CA, USA) was first introduced in 1999, as an esthetic alternative to orthodontic fixed appliances. Invisalign^®^ system uses a CAD/CAM technology to assist technicians and dentists in the creation of a a treatment plan that relies on customized aligners designed to move teeth in short intervals of time.^1^

Initially, Invisalign^®^ emerged as an option for low complexity orthodontic treatments, for example, anterior dental crowding.^2^ Over the last years, there was a notorious evolution. Change of aligner manufacturing material to SmartTrack^®^, different protocols and attachments were developed to assist treatment of difficult malocclusions. Regarding the available scientific evidence^2^, there is not a clear indication of which cases can or cannot be treated with Invisalign^®^. However, a shift on the complexity of malocclusions being treated with this system can be observed over the last years. 

The pioneer studies on Invisalign^®^ were focused on materials, technical aspects, outcomes comparison based on Peer Assessment Rating (PAR) scores, and case reports.^3^ ^-^ ^5^ It is important to notice that scientific evidenced also evolved as studies started to focus on the primary goal of any new treatment. Kravitz et al^1^ were the first ones to evaluate the accuracy of tooth movement in patients treated with clear aligners, and since then many other authors have followed.^6^ ^-^ ^12^

On January 2013, there was a global change in the manufacturing material of Invisalign^®^ aligners, when EX30^®^ (Polyethylene Terephthalate Glycol, or PETG) was replaced by SmartTrack^®^ (Polyurethane), which is in current use.^13^ ^,^ ^14^ According to Invisalign^®^, SmartTrack^®^ material would improve control and predictability of tooth movements due to its flexibility.

Thus, the purpose of the present clinical retrospective study is to evaluate the predictability of treatment with Invisalign^®^ aligners, regarding rotational, mesio-distal tip and buccal-lingual movements; and, also, to compare the predictability of both Invisalign^®^ materials (EX30^®^ x SmartTrack^®^) in achieving the prescribed movement.

## MATERIAL AND METHODS

This was a retrospective observational study, approved by the São Paulo University, under the number 2.865.423. This investigation was characterized as a study employing a convenience sampling strategy, wherein the SmartTrack^®^ sample size was three times greater than the EX30^®^ sample. Such a sampling approach is instrumental in addressing pragmatic considerations associated with participant recruitment. To ascertain a statistical power of 80% and adhere to an alpha level of 0.05, with the objective of detecting a minimum effect size of 0.05, the calculations indicated a requisite minimum of 43 participants for the EX30^®^ group and 127 participants for the Smart-Track^®^ group.

All patients were treated with Invisalign^®^ Comprehensive, with aligner change every two weeks. Inclusion criteria were: (1) nonextraction Invisalign^®^ treatments, (2) no midcourse corrections, (3) no combined treatment with fixed appliances or any other auxiliary appliance. Exclusion criteria were patients who did not complete treatment with the first sequence of aligners, who presented autoimmune diseases, pregnant and lactating women, and those whose final digital scans exceeded 45 days post-treatment. Patients in need of orthognathic surgery, orthodontic extractions, resolution of dental crowding superior to 5 mm, temporary anchorage devices or presence of edentulous space were also excluded.

Invisalign^®^ treatments were prescribed by two highly experienced orthodontists (E.K.C.R, J.A.M.M.), and conducted at their own private offices. In this study, we evaluated tooth movement accuracy of the first set of aligners, although additional aligners were prescribed to finalize treatment. All teeth were assessed via three sets of digital models throughout treatment: model 1 - initial; model 2 - predicted tooth position, and model 3 - achieved after using the first set of aligners. All digital models were exported in Standard Triangle Language (.STL) format. 

For each tooth, the degree of rotation, mesio-distal and buccal-lingual tip were measured. Then, the predicted delta (the variation between the initial and final planned models) and the achieved delta (the variation between the initial and the final achieved models) were calculated. EX30^®^ delta and SmartTrack^®^ delta were the variation between predicted and achieved deltas.

**Figure 1:**
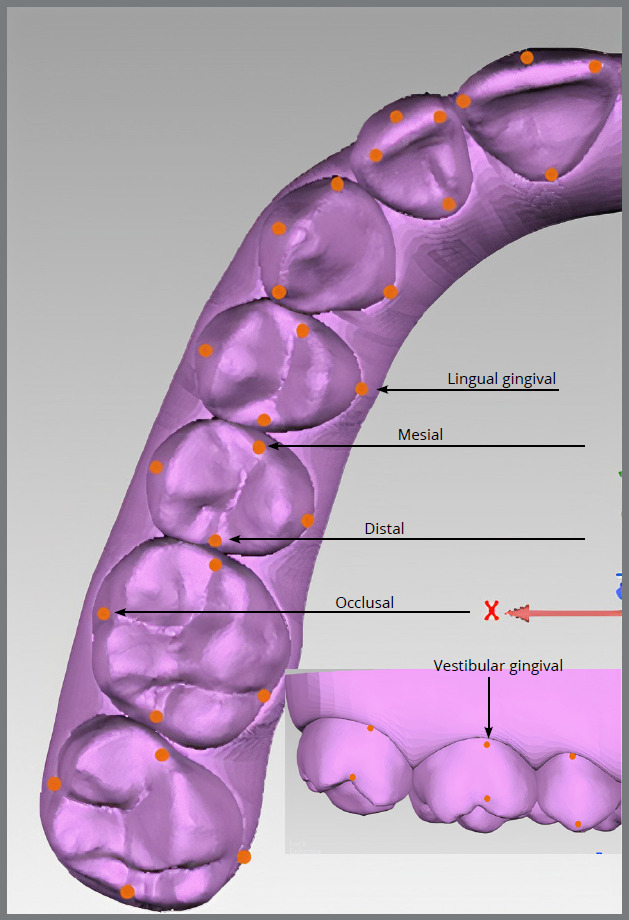
Lingual gingival, mesial, distal, occlusal and vestibular gingival points marked on teeth of initial models.

**Figure 2:**
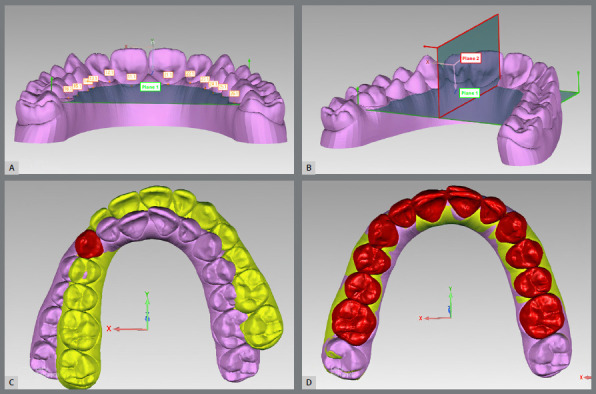
A) Reference plane created by the best adjustment of the lingual gingival points of all teeth. B) Plane 2 was created perpendicular to the XZ plane and to the reference plane. C) Model 1 in purple and Model 2 in yellow. To transfer the canine’s points from 1 to 2, best fit alignment of this tooth was performed, and five points were copied from the former to the latter. D) To transfer the reference plane and Plane 2, best fit alignment of all teeth was performed, then copied from Model 1 to Model 2 and subsequently to Model 3.

Following the previously described methodology of Santos et al.,^15^ .STL models were imported into Geomagic Control^®^ (3D Systems, Rock Hill, SC, USA) software. Five points (lingual gingival, mesial, distal, occlusal, and vestibular gingival) were marked on each tooth of Model 1 (Fig 1). As angular measurements require a reference plane, it was defined in Model 1 as the best adjustment between the lingual gingival points of all teeth, except second molars, and named Plane 1 (Fig 2A). A second plane, perpendicular to the reference plane and named Plane 2, was also created (Fig 2B). The Cartesian space (XYZ) was then reoriented so that the XY plane coincided with Plane 1 and the YZ plane coincided with Plane 2. 

The points marked on Model 1 were transferred to Model 2, as illustrated in Figure 2C. Planes 1 and 2 were also transferred (Fig 2D). The Cartesian space (XYZ) was reoriented in the final models in the same way as described for the initial model.

**Figure 3:**
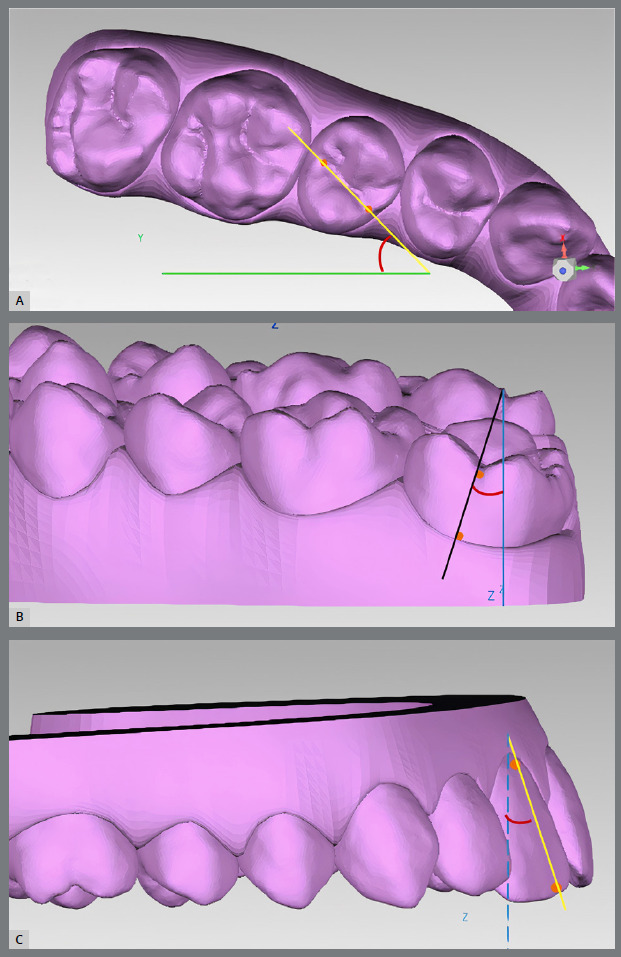
A) The line created by mesial and distal points is shown in yellow, and the line parallel to Y axis, in green. The rotation was defined by the angle between these two lines. B) The line formed by occlusal and vestibular gingival points is in black, and Z axis, in blue. The angulation was obtained in relation to the YZ plane. C) The line formed by the occlusal and buccal gingival points is shown in yellow. The blue line represents the Z axis. The inclination was obtained in relation to the XZ plane.

Rotation was defined as the angle between the line formed by the mesial and distal points of each tooth and the Y axis (Fig 3A). For the measurement of mesio-distal and buccal-lingual tip (Figs 3B and 3C), in order to fully capture the movement and not just one vector component, it was necessary to reorient the Cartesian space of each tooth with the aid of a rotational matrix, as described by Santos et al.^15^ and Huanca et al.^1^ ^6^

The degree of rotation, mesial-distal tip and buccal-lingual tip was measured for each tooth in models 1, 2 and 3. Then, the predicted delta (the variation of tooth position between models 2 and 1) and the achieved delta (the variation of tooth position between models 3 and 1) were calculated. Thus, the discrepancy for teeth treated with SmartTrack^®^ (SmartTrack^®^ delta: predicted delta - achieved delta) and with EX30^®^ (EX30^®^ delta: predicted delta - achieved delta) were also calculated.

## STATISTICAL ANALYSIS

The outcome variables were the SmartTrack^®^ delta and EX30^®^ delta, considering rotation, mesio-distal and buccal-lingual tip. The discrepancy was calculated per tooth, considering the differences in module between the predicted and achieved positions. As teeth were clustered in patients, a multilevel approach was used. Variables were submitted to Shapiro-Francia and Levene tests, to assess normality and homogeneity of variance of these variables. Initially, comparisons between EX30^®^ delta and SmartTrack^®^ delta were done by multilevel linear regression with robust variance, by groups of teeth (incisors, canines, premolars and molars).

Posteriorly, the influence of variables on these outcomes were evaluated by multilevel linear regression with robust variance, including the technique. The explanatory variables were teeth group, predicted movement, dental arch, presence of attachment, number of aligners, age and sex. Univariate analysis was conducted, and all variables with *p*-value lower than 0.05 were included. Analysis was performed using Stata 15.0 (Stata Corp, College Station, USA), and level of significance was set at 0.05.

## RESULTS

The sample consisted of 1298 teeth from 56 adult patients (17 male, 39 female) with mean age of 33 years. The distribution of malocclusions was as follows: 26 Class I, 26 Class II and 4 Class III. The average number of aligners per treatment was 24, with mean treatment time of 11.2 months. The sample was classified according to the material used for Invisalign’s aligners: EX30^®^ group (Polyethylene Terephthalate Glycol, or PETG) (n = 301 teeth) - aligners manufactured until January 27^th^ 2013; SmartTrack^®^ group (polyurethane) (n = 997 teeth) - aligners manufactured from January 28^th^ 2013 until the present date. The types of movement evaluated were rotation, mesio-distal tip, and buccal-lingual tip. 

The accuracy outcomes for EX30^®^ and SmartTrack^®^ groups were compared according to types of tooth movement and teeth group (*p* > 0.05) (Table 1). The ideal achievement would be to reach zero discrepancies between predicted and achieved tooth positions.

**Table 1:** Mean discrepancy between predicted and achieved tooth positions for EX30^®^ and SmartTrack^®^ groups.

Teeth group	EX30^®^ delta		SmartTrack^®^ delta		p value *
	n	Mean (SD)	n	Mean (SD)	
Rotation					
Incisors	104	4.52 (4.12)	337	4.32 (4.38)	0.760
Canines	52	5.50 (5.74)	170	6.45 (6.40)	0.457
Premolars	95	4.59 (3.86)	323	4.75 (5.48)	0.820
Molars	50	2.30 (2.34)	167	2.27 (3.37)	0.933
Total	301	4.34 (4.23)	997	4.48 (5.15)	
Mesio-Distal tip					
Incisors	104	2.49 (2.39)	337	2.70 (2.44)	0.479
Canines	52	2.82 (1.97)	170	3.60 (3.27)	0.097
Premolars	95	2.52 (2.10)	323	3.01 (2.66)	0.178
Molars	50	1.61 (1.88)	167	2.04 (1.81)	0.159
Total	301	2.41 (2.17)	997	2.84 (2.62)	
Buccal-Lingual tip					
Incisors	104	2.46 (2.58)	337	3.68 (3.50)	0.007
Canines	52	1.91 (1.73)	170	2.93 (2.76)	0.007
Premolars	95	2.61 (2.43)	323	2.96 (2.93)	0.385
Molars	50	1.78 (1.82)	167	2.32 (2.57)	0.197
Total	301	2.30 (2.30)	997	3.09 (3.09)	

SD = Standard deviation.

* calculated by multilevel regression analysis with robust variance.

Table 1 presents SmartTrack^®^ delta, EX30^®^ delta and their comparison by multilevel linear regression. When analyzing the accuracy of tooth movements regarding rotation and mesio-distal tip, no differences were found between EX30^®^ and SmartTrack^®^ groups (*p* ˃ 0.05). For buccal-lingual tip, however, EX30^®^ aligners seem to be slightly more accurate for moving incisors and canines than SmartTrack^®^ (Table 1).

Table 2 presents the mean values of predicted tooth movement for EX30^®^ and SmartTrack^®^ groups. 

**Table 2:** Mean values of predicted tooth movement for the EX30 and SmartTrack

Teeth group	EX30^®^		SmartTrack^®^	
	n	Mean (SD)	n	Mean (SD)
Rotation				
Incisors	104	10.81 (14.06)	337	8.71 (10.16)
Canines	52	8.64 (8.45)	170	10.69 (8.94)
Premolars	95	7.48 (7.26)	323	7.22 (7.46)
Molars	50	2.99 (3.94)	167	3.56 (5.19)
Total	301	8.09 (10.30)	997	7.70 (8.70)
Mesio-Distal tip				
Incisors	104	3.61 (3.51)	337	3.81 (3.52)
Canines	52	2.73 (2.31)	170	4.71 (3.96)
Premolars	95	1.81 (2.01)	323	3.75 (3.21)
Molars	50	1.45 (1.37)	167	2.01 (2.23)
Total	301	2.53 (2.73)	997	3.64 (3.41)
Buccal-Lingual tip				
Incisors	104	4.46 (4.38)	337	5.58 (4.83)
Canines	52	2.75 (2.79)	170	4.48 (3.77)
Premolars	95	2.67 (2.93)	323	4.38 (4.24)
Molars	50	1.32 (1.68)	167	2.21 (3.25)
Total	301	3.08 (3.51)	997	4.44 (4.37)

SD = Standard deviation.

Tables 3, 4 and 5 analyze the influence of variables on the predictability of tooth movement with aligners. Both teeth moved with EX30^®^ and with SmartTrack^®^ were included in the analysis. Groups were now divided according to tooth and patient-related variables. The tooth-related variables were: material (EX30^®^/SmartTrack^®^), predicted movement (quantity of rotation/mesio-distal tip/buccal-lingual tip), teeth group (incisors/canines/pre-molars/molars), dental arch (upper/lower), and presence of attachment (yes/no). The patient-related variables were: number of aligners (up to 14 or higher), age (quantity of years), and sex (male/female). 

**Table 3:** Multilevel linear regression analysis of rotation movements.

Explanatory variables	Unadjusted Coefficient (SE)	p *	Adjusted Coefficient (SE)	p *
TOOTH-RELATED VARIABLES				
Material (ref.: EX30)				
SmartTrack	0.12 (0.51)	0.818	-0.12 (0.49)	0.811
Predicted rotation (quant. variable)	0.36 (0.09)	<0.001	0.35 (0.09)	<0.001
Teeth group (Ref.: incisors)				
Canines	1.86 (0.51)	<0.001	1.26 (0.41)	0.002
Premolars	0.35 (0.40)	0.382	0.64 (0.36)	0.079
Molars	-2.07 (0.31)	<0.001	-0.07 (0.34)	0.842
Dental arch (ref.: upper)				
Lower	0.26 (0.42)	0.539		**
Presence of attachment (ref.: no)				
Yes	2.06 (0.33)	<0.001	0.56 (0.51)	0.273
PATIENT-RELATED VARIABLES				
Number of aligners (ref.: up to 14)				
More than 14	1.15 (0.35)	0.001	-0.03 (0.27)	0.903
Age (quant. variable)	0.02 (0.03)	0.496		**
Sex (ref.: female)				**
Male	-0.24 (0.47)	0.608		

SE = Standard error.

* calculated by multilevel linear regression analysis with robust variance.

** variable not included in the multiple model.

**Table 4:** Multilevel linear regression analyses of mesio-distal tip.

Explanatory variables	Unadjusted Coefficient (SE)	p *	Adjusted Coefficient (SE)	p *
TOOTH-RELATED VARIABLES				
Material (ref.: EX30)				
SmartTrack	0.41 (0.25)	0.096	0.004 (0.173)	0.981
Predicted mesio-distal tip (quant. variable)	0.40 (0.04)	<0.001	0.40 (0.04)	<0.001
Teeth group (Ref.: incisors)				
Canines	0.77 (0.24)	0.002	0.59 (0.22)	0.007
Premolars	0.28 (0.20)	0.154	0.47 (0.18)	0.011
Molars	-0.69 (0.19)	<0.001	0.05 (0.18)	0.768
Dental arch (ref.: upper)				
Lower	0.19 (0.16)	0.246		**
Presence of attachment (ref.: no)				
Yes	0.54 (0.28)	0.052	-0.03 (0.13)	0.814
PATIENT-RELATED VARIABLES				
Number of aligners (ref.: up to 14)				
More than 14	0.54 (0.28)	0.052	-0.02 (0.21)	0.915
Age (quant. variable)	0.04 (0.01)	0.001	0.01 (0.01)	0.138
Gender (ref.: female)				**
Male	-0.46 (0.27)	0.088		

SE = Standard error.

* calculated by multilevel linear regression analysis with robust variance.

** variable not included in the multiple model.

Table 3 presents the influence of tooth-related and patient-related variables among all teeth included in this study over the accuracy of rotation movements. In reference to the manufacturing material, there were no differences between EX30^®^ and SmartTrack^®^ groups (*p*=0.81). The quantity of predicted rotation presented an influence on the accuracy of tooth movements (*p*<0.001). For each additional degree planned for rotation movements, there was an increase of 0.35° in the discrepancy between predicted and achieved tooth positions, meaning that inaccuracy increased, on average, 0.35°. Table 3 also shows that rotating canines was less accurate than rotating incisors (*p*=0.002), on average, 1.26°. There were no differences in the accuracy of rotating lower or upper teeth (*p*˃0.05). Presence or absence of attachments, age and sex did not influence the discrepancy between predicted and achieved tooth positions. Patients treated with up to 14 aligners did not achieve more accurate rotation movements than that of patients treated with more than 14 aligners (*p*˃0.05).

Table 4 presents the influence of tooth and patient-related variables over the accuracy of mesio-distal tip. In reference to the manufacturing material, there were no differences between EX30^®^ and SmartTrack^®^ groups (*p*=0.98). The quantity of predicted mesio-distal tip presented an influence on the accuracy of tooth movements (*p*<0.001). For each additional degree planned for mesio-distal tip, there was an increase of 0.4° in the discrepancy between predicted and achieved tooth positions, meaning that the inaccuracy increased, on average, 0.4°. Performing mesio-distal tip of canines and premolars is less accurate than performing mesio-distal tip of incisors, on average, 0.59° and 0.47° respectively. There were no significant differences in the accuracy of lower and upper arches (*p*˃0.05). Presence or absence of attachments, age and sex did not influence the predictability. Patients treated with up to 14 aligners did not achieve more accurate mesio-distal tip than patients treated with more than 14 aligners (*p*˃0.05).

Table 5 presents the influence of tooth and patient-related variables over the accuracy of buccal-lingual tip. Regarding the manufacturing material, no differences were found between EX30^®^ and SmartTrack^®^ groups (*p*=0.26). The quantity of predicted buccal-lingual tip influenced the accuracy of tooth movements (*p*<0.001). For each additional degree planned, the inaccuracy increased, on average, 0.41°. No differences were found in the accuracy for inclination of lower or upper teeth (*p*˃0.05). Presence or absence of attachments, age and sex did not influence the discrepancy between predicted and achieved tooth positions.

**Table 5:** Multilevel linear regression analysis of buccal-lingual tip.

Explanatory variables	Unadjusted Coefficient (SE)	p *	Adjusted Coefficient (SE)	p *
TOOTH-RELATED VARIABLES				
Material (ref.: EX30)				
SmartTrack	0.80 (0.26)	0.002	0.20 (0.18)	0.260
Predicted buccal-lingual tip (quant. variable)	0.41 (0.05)	<0.001	0.41 (0.06)	<0.001
Teeth group (Ref.: incisors)				
Canines	-0.70 (0.29)	0.016	-0.19 (0.26)	0.469
Premolars	-0.49 (0.33)	0.139	0.10 (0.29)	0.736
Molars	-1.20 (0.34)	<0.001	0.17 (0.31)	0.589
Dental arch (ref.: upper)				
Lower	-0.17 (0.21)	0.413		**
Presence of attachment (ref.: no)				
Yes	0.03 (0.26)	0.909	-0.05 (0.21)	0.801
PATIENT-RELATED VARIABLES				
Number of aligners (ref.: up to 14)				
More than 14	0.95 (0.27)	<0.001	0.10 (0.19)	0.594
Age (quant. variable)	0.05 (0.02)	0.003	0.02 (0.01)	0.120
Sex (ref.: female)				**
Male	-0.10 (0.33)	0.761		

SE = Standard error.

* calculated by multilevel linear regression analysis with robust variance.

** variable not included in the multiple model.

## DISCUSSION

The purpose of this clinical retrospective study was to evaluate the predictability of treatment with Invisalign^®^ aligners in respect of rotational, mesio-distal and buccal-lingual tip movements; and, also, to compare the predictability of both Invisalign^®^ materials (EX30^®^ x SmartTrack^®^) in achieving the prescribed movement. Although Invisalign’s aligners are not fabricated with PETG anymore, this material is still among the commonly used, along with polyurethane and polyester.^14^

One of the major challenges to assess predictability is the use of an appropriate methodology. Some studies focus on evaluating the quality of the orthodontic finishing by using the Objective Grading System,^17^ or by evaluating parameters such as overbite and overjet,^10^ ^,^ ^17^ providing general aspects regarding accuracy. For studies that intend to investigate more precise aspects, such as the amount of movement that a specific tooth will undergo, the lack of stable structures for superimposing digital models has been a major problem.

To try to solve this issue, some studies have chosen to superimpose the posterior teeth for situations in which only anterior teeth would be moved,^1^ or to superimpose molars when a small movement of these teeth was prescribed.^18^ However, superimposing on the posterior teeth restricts analysis only to anterior teeth. Moreover, even in situations in which posterior teeth movement was not planned, the possibility of their movement can not be refuted. Some authors tried to minimize the problem via global alignment of the two analyzed models,^6^ ^,^ ^7^ which is similar to what was performed in the present study.

To the best of our knowledge, this is the first study to assess tooth movement predictability with aligners using a previously validated method.^15^ Although this method has been shown to have limited validity for mesio-distal tip and buccal-lingual tip of specific teeth, it was applied to calculate the predicted and achieved movements in all teeth, as, by using the same technique, the error maintenance principle tends to minimize the problem. In other words, if both deltas are measured by the same method, any presumed error will likely be kept in the same proportion for both measurements, making the comparison valid.

Regarding the comparison of tooth movement accuracy of EX30^®^ x SmartTrack^®^, differences were found for buccal-lingual tip of incisor and canines. For these teeth groups, EX30^®^ performed mesio-distal tip more accurately than SmartTrack^®^. Mechanical properties of thermoplastic materials used for clear aligners may, therefore, play an important role achieving specific movements. 

The available scientific evidence acknowledges that the two assessed materials have the specified characteristics for orthodontic aligners, such as: biocompatibility, transparency, low hardness, good elasticity, resilience to storage in artificial saliva.^19^ ^,^ ^20^ Condo et al.^13^ conducted an *in vitro* comparative study that analyzed structural properties of EX30^®^ and SmartTrack^®^ aligners, and observed that the latter demonstrated better adaptability to the dental arch and greater consistency on application of orthodontic forces. Tamburrino et al.^20^ investigated mechanical properties of three thermoplastic polymers commonly used for aligner manufacturing and concluded that Duran^®^, designated by the manufacturer as PETG, presented higher elastic modules (thus higher stiffness) after thermoforming. In other others, the drawn material is stronger and stiffer than before. SmartTrack^®^ meets the necessary requirements for an orthodontic aligner, while also providing comfort for the patient. However, we may hypothesize that a stronger and stiffer material such as EX30^®^ (PETG) can achieve more accurately buccal-lingual tip in anterior teeth, as demonstrated in this study.

Although buccal-lingual accuracy of EX30^®^ seems to be only slightly higher than SmartTrack^®^ for incisors and canines, the planned movement in the present study was moderate (Table 2). The multilevel linear regression analysis demonstrated that tooth movement predictability tends to worsen as a greater amount of movement is planned (Table 4). Therefore, larger requests of movement may potentially lead to higher differences between groups.

Tables 3, 4 and 5 present the influence of variables on tooth movement predictability with aligners. For these analyses, multilevel regression analyses assessed the data from all teeth included in this study, and groups were now divided according to tooth and patient-related variables. The results have shown that the greater the quantity of planned tooth movement, the greater will be the difference between predicted and achieved tooth positions. For instance, for each additional degree planned for rotation movements, accuracy decreases by 0.35°, and decreases by 0.40° and 0.41° for mesio-distal tip and buccal-lingual tip, respectively. These findings are in agreement with other studies.^1^ ^,^ ^11^ Kravitz et al.^1^ found that, for canines, rotation schedules > 15° were less predictable than <15°; while Simon et al.^11^ found that, for premolars, rotation schedules > 15° were less predictable than >10° and <15°.

It can be noticed that the molar group with unadjusted coefficient in Table 3 was, on average, 2° more accurate for rotation movements when compared to the incisor group (*p*˂0.001), and the same molar group with adjusted coefficient did not present a statistically significant difference (*p*=0.84). In groups with a low demand of tooth movement, which is the case of the molar groups in our sample, there was a “false impression” of accuracy. As observed in Table 2, the mean values of predicted tooth movement for rotation in the molar group was 2.99°, as opposed to the 10.81° for the incisor group, and 8.64° for the canines. Since there was a correlation among quantity of predicted movement and accuracy rates, it is mandatory to consider the adjusted coefficient when analyzing the results. Otherwise, the comparison among groups would be based on biased outcomes. That finding is also true for mesio-distal tip and buccal-lingual tip.

In the multilevel linear regression analysis described on Tables 3 and 5, it can be noticed that rotating canines is less accurate than rotating incisors (1.26°, on average); and performing mesio-distal tip of canines and premolars is less accurate than performing mesio-distal tip of incisors (0.59° and 0.47°, on average, respectively). It must be kept in mind that the amount of inaccuracy will vary depending on the amount of planned movement, and that the values of 1.26°, 0.59° and 0.47° are related to the amount of planned movement on the current sample. 

Regarding rotation, the study of Charalampakis et al.^18^ also demonstrated the difficulty in rotating canines, which is probably related to anatomy, as it is more challenging to rotate a rounded tooth like a canine than a rectangular one, like an incisor. In respect of mesio-distal tip, the fact that moving incisors is more accurate than moving canines and premolars is probably related to the higher flexibility that aligners present at extremities, reason why they seem to respond less on posterior regions.^6^ A lower accuracy in molar movement was not observed in the present study, possibly due to the limited request for molar movement in the current sample (see Table 2). 

## CONCLUSION

» EX30 aligners reached higher accuracy for buccal-lingual tip in anterior teeth.
» The total amount of planned movement had a significant impact on the accuracy rates, with a decrease in accuracy for every additional degree.
» Rotation of incisors was more accurate than the rotation of canines. Similarly, the mesio-distal tip of incisors was also more predictable than the angulation of canines and premolars.
